# Isolation Rate, Antimicrobial Resistance, Virulence-Associated Genes, and Multilocus Sequence Types of *Klebsiella pneumoniae* Isolated from Cattle with Bovine Respiratory Disease in Henan Province, China

**DOI:** 10.3390/vetsci13101029

**Published:** 2026-09-29

**Authors:** Hongfei Shi, Hongyue Zhai, Yuli Xie, Tong Xiao, Shuran Yang, Zhaoxu Chen, Yilin Bai

**Affiliations:** 1 Henan Provincial Engineering and Technology Center of Animal Disease Diagnosis and Integrated Control, Henan Key Laboratory of Insect Biology, Nanyang Normal University, Nanyang 473061, China; zhaihongyue163@163.com (H.Z.); yulixie87@163.com (Y.X.); wy123456781005@126.com (T.X.); yangshuran624@163.com (S.Y.); 16696496182@163.com (Z.C.); 2 Key Laboratory of Innovative Utilization of Local Cattle and Sheep Germplasm Resources (Co-Construction by Ministry and Province), Ministry of Agriculture and Rural Affairs, Zhengzhou University, Zhengzhou 450001, China

**Keywords:** *Klebsiella pneumoniae*, cattle, bovine respiratory disease, antimicrobial resistance, virulence-associated genes, multilocus sequence typing

## Abstract

Bovine respiratory disease (BRD) is a major cause of morbidity and mortality in cattle, and *Klebsiella pneumoniae* (*K. pneumoniae*) has emerged as an opportunistic pathogen that can colonize the bovine respiratory tract. However, epidemiological data on *K. pneumoniae* from cattle with BRD in Henan Province, central China, remain scarce. This study aimed to investigate the isolation rate, antimicrobial resistance profiles, resistance genes, virulence-associated genes, and genetic diversity of *K. pneumoniae* from BRD-affected calves in Henan. From 2021 to 2026, deep nasal swabs were collected once from 517 calves with clinical signs of BRD (Wisconsin score ≥5) across 38 farms in 13 cities over six years. A total of 87 *K. pneumoniae* isolates were obtained, giving an overall isolation rate of 16.8% (87/517), ranging from 13.8% to 22.6% across five geographic regions. Antimicrobial susceptibility was determined by disk diffusion using CLSI VET01S and CLSI M100-S26 criteria for 15 antimicrobials belonging to six classes. Resistance and virulence-associated genes were screened by PCR, and multilocus sequence typing (MLST) was performed. MLST revealed 34 sequence types (STs), with ST950 being the most prevalent (6.9%), followed by ST262, ST187, and ST306. Antimicrobial resistance was highest against ampicillin (94.3%), sulfisoxazole (79.3%), cefoxitin (73.6%), cefazolin (70.1%), and enrofloxacin (67.8%). Multidrug resistance (resistance to ≥3 classes) was observed in 96.6% (84/87; 95% CI 90.3–98.8%) of isolates. The most common resistance genes were *bla*TEM (72.4%), *sul1* (39.1%), *bla*SHV (37.9%), and *qnrB* (14.9%). After Firth’s penalized logistic regression with farm-level cluster-robust standard errors and Benjamini–Hochberg FDR correction, no resistance gene was significantly associated with phenotypic resistance (all q > 0.05). Virulence-associated genes *fimH*, *mrkD*, *uge*, *wabG*, *entB*, *iutA*, and *ureA* were highly prevalent (>90%), whereas hypervirulence markers (*rmpA* and *rmpA2*) were absent. The most common virulence-associated gene combination (eight genes) was present in 35.6% of isolates. These findings indicate high genetic diversity, widespread multidrug resistance, and a broad virulence-associated gene repertoire among *K. pneumoniae* isolated from cattle with BRD in Henan. Because no healthy comparison group or etiological confirmation was included, the study does not establish *K. pneumoniae* as the causative agent of BRD. The high resistance rates to β-lactams, sulfonamides, and quinolones highlight the need for prudent antimicrobial use. Florfenicol showed the lowest in vitro resistance rate among the tested agents; however, clinical efficacy, treatment outcome, and safety were not evaluated. Continuous surveillance and molecular monitoring are essential to manage this pathogen in cattle populations.

## Simple Summary

*Klebsiella pneumoniae* is an opportunistic bacterium that can colonize the respiratory tract of cattle and has been isolated from calves with bovine respiratory disease (BRD). We collected deep nasal swabs once from 517 calves with BRD on 38 farms in Henan, China, over six years (2021–2026). *K. pneumoniae* was isolated from 16.8% of the samples. The isolates showed high genetic diversity (34 sequence types) and widespread multidrug resistance, especially to β-lactams, sulfonamides, and quinolones. Of the isolates, 96.6% were multidrug-resistant. Many resistance and virulence-associated genes were detected, but classical hypervirulence markers were absent. Because no healthy control calves or causality experiments were included, these findings do not establish *K. pneumoniae* as the causative agent of BRD. Prudent antibiotic use and ongoing surveillance are essential to control this pathogen and protect cattle health.

## 1. Introduction

Bovine respiratory disease (BRD), also known as bovine bronchopneumonia, is a major cause of morbidity and mortality in intensively managed feedlot and dairy calf production systems, although estimates vary by production system, age, management, and geographic region [1,2,3,4]. BRD is a multifactorial complex involving management stress, infectious agents, and antimicrobial exposure, and the bacterial pathogens routinely isolated are frequently opportunistic [5,6,7,8]. Recent studies in intensively managed beef cattle, such as Masebo et al. (2024), have documented a high burden of respiratory disease, extensive antimicrobial treatment, and persistent pulmonary lesions at slaughter, emphasizing the difficulties associated with antimicrobial management of BRD [9]. Longitudinal surveillance studies, such as Dini et al. (2024), further illustrate how infectious exposure can change over time in relation to farm management and biosecurity conditions [10].

*Klebsiella pneumoniae* (*K. pneumoniae*) is a Gram-negative opportunistic pathogen that can colonize the respiratory or intestinal tracts of both humans and animals [11,12]. It has been associated with a range of infectious diseases, including mastitis, pneumonia, bacteremia, liver abscess, urinary tract infection, arthritis, and meningitis, thereby posing a threat to human health and livestock husbandry [11]. *K. pneumoniae* isolates in cattle have been reported in many countries [13,14,15]. In China, numerous studies have demonstrated that *K. pneumoniae* can infect dairy cows and cause mastitis [16,17,18]. Some studies have suggested that *K. pneumoniae* is associated with BRD in regions such as Hebei province and Chongqing City, China [6,19]. To the best of our knowledge, only one study has reported the isolation of *K. pneumoniae* from cows in Henan Province in 2017, but that paper did not report the health status of the cows or the sample types [20]. Therefore, the epidemiological patterns of *K. pneumoniae* in cattle herds in Henan in recent years remain unclear. Considering that Henan borders Hebei Province and more than 3,500,000 cattle have been kept in Henan province in central China [21], it is essential to characterize *K. pneumoniae* isolates obtained from cattle with BRD.

Numerous studies have examined the diversity of *K. pneumoniae* in both humans and animals. Multilocus sequence typing (MLST) assigns sequence types (STs) on the basis of allelic profiles of seven housekeeping loci; isolates with identical allelic profiles are assigned the same ST [22]. In humans in China, many STs have been identified by MLST among *K. pneumoniae* isolates [23,24]. Another investigation identified two STs using MLST in pigs in China [25]. These findings suggest that genetic diversity among animal *K. pneumoniae* is widespread. To our knowledge, no studies have yet explored the predominant STs of *K. pneumoniae* strains associated with BRD in Henan province, China, leaving a gap in the available molecular epidemiological data.

Antimicrobial therapy continues to be the cornerstone for both the prevention and treatment of BRD. In China, a wide range of antimicrobials have been approved for this indication, including β-lactams, tetracyclines, sulfonamides, quinolones, and aminoglycosides. Nevertheless, the extensive use of these agents has fostered the emergence of multidrug resistance through selection of acquired resistance genes, chromosomal mutations, mobile genetic elements, and clonal dissemination, progressively diminishing their clinical efficacy [6,19,26,27]. Although nationwide programs for the monitoring and surveillance of antimicrobial resistance in animals have been in place in China for many years, information on antibiotic resistance in *K. pneumoniae* derived from cattle remains scarce. The resistance mechanisms of *K. pneumoniae* are tightly linked to specific resistance genes; for instance, *blaIMP*, *blaVIM*, *blaOXA-48*, *blaNDM*, *blaKPC*, *blaDHA*, *blaFOX*, *blaCTX-M-2*, *blaSHV*, and *blaTEM* are regarded as the major genes underlying β-lactam resistance [28,29], while *sul1* and *sul2* mediate resistance to sulfonamides [29]. The potential transfer of these resistance determinants to other bacteria, including those in the human respiratory microbiota, is a public-health concern [30]; however, the present study did not investigate transmission.

Virulence-associated genes serve as the essential determinants of bacterial pathogenicity. Typically, *K. pneumoniae* possesses a range of virulence factors—such as fimbriae, capsular polysaccharides, lipopolysaccharides, and siderophores—that are encoded by various virulence-associated genes, including *fimH*, *rmpA*, *rmpA2*, *uge*, *wabG*, *iutA*, and *iroN* [31]. According to previous reports, the expression of these virulence-associated genes is closely linked to the infectivity and pathogenicity of *K. pneumoniae* [29]. However, detection of a virulence-associated gene by PCR does not demonstrate its expression or establish pathogenicity.

To the best of our knowledge, no prior research has investigated the antimicrobial susceptibility and genetic diversity of *K. pneumoniae* originating from cattle with BRD in Henan province, China. The objective of this study was to characterize *K. pneumoniae* isolates obtained from calves with BRD in Henan Province, including isolation rate, antimicrobial susceptibility, resistance genes, virulence-associated genes, and MLST sequence types.

## 2. Materials and Methods

### 2.1. Sample Collection, Bacterial Isolation, and Identification

From 2021 to 2026, a total of 517 calves with clinical BRD were enrolled in this study. The calves ranged in age from 1 to 6 months (median 3 months; IQR 2–4 months). The sex distribution was 270 males (52.2%) and 247 females (47.8%). Production types included intensive (10 farms), semi-intensive (17 farms), and backyard (11 farms) systems. Information on antimicrobial exposure before sampling was recorded: 14 farms (36.8%) reported antimicrobial use within 14 days before sampling, 22 farms (57.9%) reported no use, and 2 farms (5.3%) had unknown status (Table S1). Farms were selected according to the following criteria: ≥20 calves, a history of BRD, and owner consent. Within each farm, calves were selected if they had a Wisconsin score ≥5, had not been sampled previously, and had no other obvious systemic disease.

According to the Wisconsin Calf Respiratory Scoring System [32], all calves presented with clinical signs corresponding to a total score of ≥5, including fever, cough, nasal discharge, ocular discharge, and ear droop. Clinical scoring was performed by trained field veterinarians. The same scoring sheet, threshold, and procedures were used throughout the six-year period. Inter-observer standardization was performed before each sampling season. No clinically healthy control calves were included in this study; this is a major limitation and is discussed below.

These calves were distributed across 38 farms in 13 cities of Henan Province. Sampling was performed annually from 2021 to 2026. The specific sampling periods, numbers of farms and cities, sample sizes, positive counts, and isolation rates for each year are summarized in Table 1 and Table S1. No calf was sampled more than once; repeated sampling of the same animal was excluded (Table 1 and Table S1). Samples were collected from deep nasal swabs. Following collection, samples were placed in sterile tubes, kept on ice, and transported to the laboratory for analysis.

For bacterial identification, samples were directly streaked onto MacConkey agar without enrichment (Beijing Solarbio Science & Technology, China) and incubated at 37 °C for 18–24 h. Up to five presumptive *K. pneumoniae* colonies per sample—small, pink, with smooth, moist surfaces and well-defined edges—were subcultured and identified by Gram staining and biochemical identification. Mixed cultures were purified by repeated streaking. Suspected *K. pneumoniae* isolates were confirmed by PCR detection using *khe* species-specific primers [28]. Briefly, genomic DNA was extracted using a commercial kit (TransGen Biotech, China) per the manufacturer’s instructions, dissolved in 100 µL of ultrapure water, and stored at −70 °C. Five randomly selected *khe*-positive isolates were sequenced to confirm the specificity of the *khe* PCR. All remaining isolates were identified by *khe* PCR following presumptive biochemical identification. One confirmed isolate per sample was stored for further analysis, using *K. pneumoniae* ATCC700603 for quality control. Selecting only one isolate per sample may underestimate within-host strain diversity; this limitation is acknowledged in the Discussion. The isolation rate was calculated as (number of positive cases/total samples) × 100%.

### 2.2. MLST Analysis of K. pneumoniae

MLST analyses of *K. pneumoniae* isolates were carried out following a previously described protocol [22]. Primers targeting seven housekeeping genes (*rpoB*, *gapA*, *mdh*, *pgi*, *phoE*, *infB*, and *tonB*) were used for PCR amplification [22]. The amplification was conducted with an annealing temperature of 50 °C for all genes, except for *gapA* (60 °C) and *tonB* (45 °C). Purified PCR products were subjected to sequencing. Sequence quality was assessed using chromatograms; low-quality ends were trimmed. Alleles and STs were assigned using the online tool at https://bigsdb.pasteur.fr/klebsiella/ [22] (accessed on 18 July 2026). All allelic profiles corresponded to previously assigned STs; no novel alleles or STs were identified.

### 2.3. Antimicrobial Susceptibility Testing

The antibiotic resistance patterns of each positive *K. pneumoniae* isolate from the samples (selected and stored in Section 2.1) were determined by disk diffusion methods according to Clinical and Laboratory Standards Institute (CLSI) guidelines (CLSI VET01S and CLSI M100-S26) [33,34]. The inhibition zones were measured and recorded. For the interpretation of inhibition zones, the antibiotic standards already included in CLSI VET01S Table 2A should be used preferentially [33]; for those not included in CLSI VET01S, refer to the standards in CLSI M100-S26 Table 2A. The exact zone diameter breakpoints (S/I/R, in mm) for every antimicrobial tested are listed in Table 2 [34].

Resistance to 15 antimicrobials belonging to six antimicrobial categories were investigated in the drug susceptibility test. Macrolides (erythromycin and azithromycin) were not included because members of the family *Enterobacteriaceae*, including *K. pneumoniae*, are intrinsically resistant to macrolides. Antimicrobials were selected because they are commonly used for BRD treatment in the region, based on information provided by farm owners. No quantitative farm-specific antimicrobial use data were systematically collected; therefore, relationships between use and resistance could not be analyzed. For each isolate–antimicrobial combination, disk diffusion was performed in three independent cultures. Discordant classifications were resolved by a fourth independent test, and the majority result was recorded. *K. pneumoniae* ATCC700603 and *Escherichia coli* ATCC 25922 were served as quality control strains. Isolates exhibiting resistance to three or more antimicrobial categories were classified as multidrug resistant (MDR) [35].

### 2.4. Detection of Resistance Genes

Genomic DNA was extracted from the bacterial isolates as described in the identification section. Conventional PCR was used to detect antibiotic resistance genes. Based on the categories of antimicrobials tested and the resistance genes most frequently reported in *K. pneumoniae* from China [19,28,31], we targeted the following: β-lactam resistance genes (*blaVIM*, *blaSHV*, *blaTEM*, *blaCTX-M-2*) [29,36,37]; sulfonamide resistance gene (*sul1, sul2,* and *sul3*) [38]; tetracycline resistance genes (*tetA*, *tetB, tetC*) [39,40,41]; phenicol resistance gene (*catI*, *cmlA*, *floR*) [42,43]; aminoglycoside resistance gene (*aadA1*, *aacC1,* and aac*C2*) [40,44]; and quinolone resistance genes (*oqxA*, *aac(6′)-Ib-cr, qnrA*, *qnrB*, *qnrS*, and *gyrA*) [20,45,46,47,48]. Primer details are listed in Table S2.

PCR was performed using an EasyTaq^®^ PCR SuperMix kit (TransGen, China). Each 20 µL reaction contained 10 µL of 2× SuperMix, 0.4 µM of each primer, and 20 ng of template DNA. The cycling protocol consisted of an initial denaturation at 94 °C for 5 min, followed by 30 cycles of 94 °C for 30 s, annealing at a primer-specific temperature for 30 s (see Table S2), 72 °C for 30 s, and a final extension at 72 °C for 10 min. Gene-positive reference strains and well-characterized clinical isolates carrying *blaTEM, blaSHV, sul1, sul2, tetC, aadA1, qnrB*, and other targets were used as positive controls. No-template controls were included in every run. PCR products were separated by electrophoresis on a 2% agarose gel (Solarbio, China) at 120 V for 60 min.

### 2.5. Detection of Virulence-Associated Genes

A panel of virulence-associated genes was screened by PCR. Targeted genes included those fimbriae synthesis-related genes (*fimH*, *mrkD*) [49,50]; lipopolysaccharide-related genes (*uge*, *wabG*) [49,51]; iron uptake system genes (*entB*, *iutA*, *iroN*, *kfu*) [29,52,53]; urease-related genes (*ureA*, *allS*) [49,54]; tellurite resistance gene (*terB*) [55]; *Hemolysin* gene (*hly*) [29]; and capsular polysaccharide synthesis and synthesis regulation related gene (*rmpA* and *rmpA2*) [55]. *K. pneumoniae* ATCC 43816 and *K. pneumoniae* NTUH-K2044 were used as positive controls for virulence-associated gene PCR assays. A no-template control was included in every PCR run. The primers, corresponding product sizes, and annealing temperatures for all virulence genes are listed in Table S3. The PCR protocols were consistent with those described in Section 2.4.

### 2.6. Statistical Analyses

Isolation rates are reported with 95% confidence intervals. Farm-level clustering was accounted for using mixed-effects logistic regression with farm as a random intercept, or cluster-robust standard errors where appropriate. To compare isolation rates across years, we used a chi-square test for heterogeneity and pairwise proportion tests with Bonferroni adjustment. A trend test was not performed because the objective was to assess whether isolation rates differed among years rather than to test for a linear trend. For genotype–phenotype analyses, 2 × 2 contingency tables were constructed for each gene–antimicrobial class pair. An isolate was considered resistant to a class if it was resistant to at least one agent within that class. Because several gene–class pairs exhibited complete or quasi-complete separation, Firth’s penalized logistic regression was used to obtain finite coefficient estimates. Farm-level clustering was accounted for by computing cluster-robust standard errors from the score function, with farm as the clustering variable. Odds ratios (ORs) with 95% confidence intervals (CIs) were derived from the Firth model. Cluster-adjusted *p*-values were obtained from the cluster-robust standard errors. Benjamini–Hochberg false discovery rate (FDR)-adjusted q-values were calculated based on the cluster-adjusted *p*-values. Associations with q < 0.05 were considered statistically significant. For gene–class pairs with complete separation or sparse data, ORs and cluster-adjusted *p*-values could not be estimated and were reported as NE (not estimable). Because the number of isolates for each ST was too small to support a robust difference analysis, this study did not perform significance testing on the character differences among different STs of *K. pneumoniae*. All analyses were performed using R version 4.5.2 (R Foundation for Statistical Computing, Vienna, Austria).

## 3. Results

### 3.1. Isolation and Identification of K. pneumoniae

**Figure 1:**
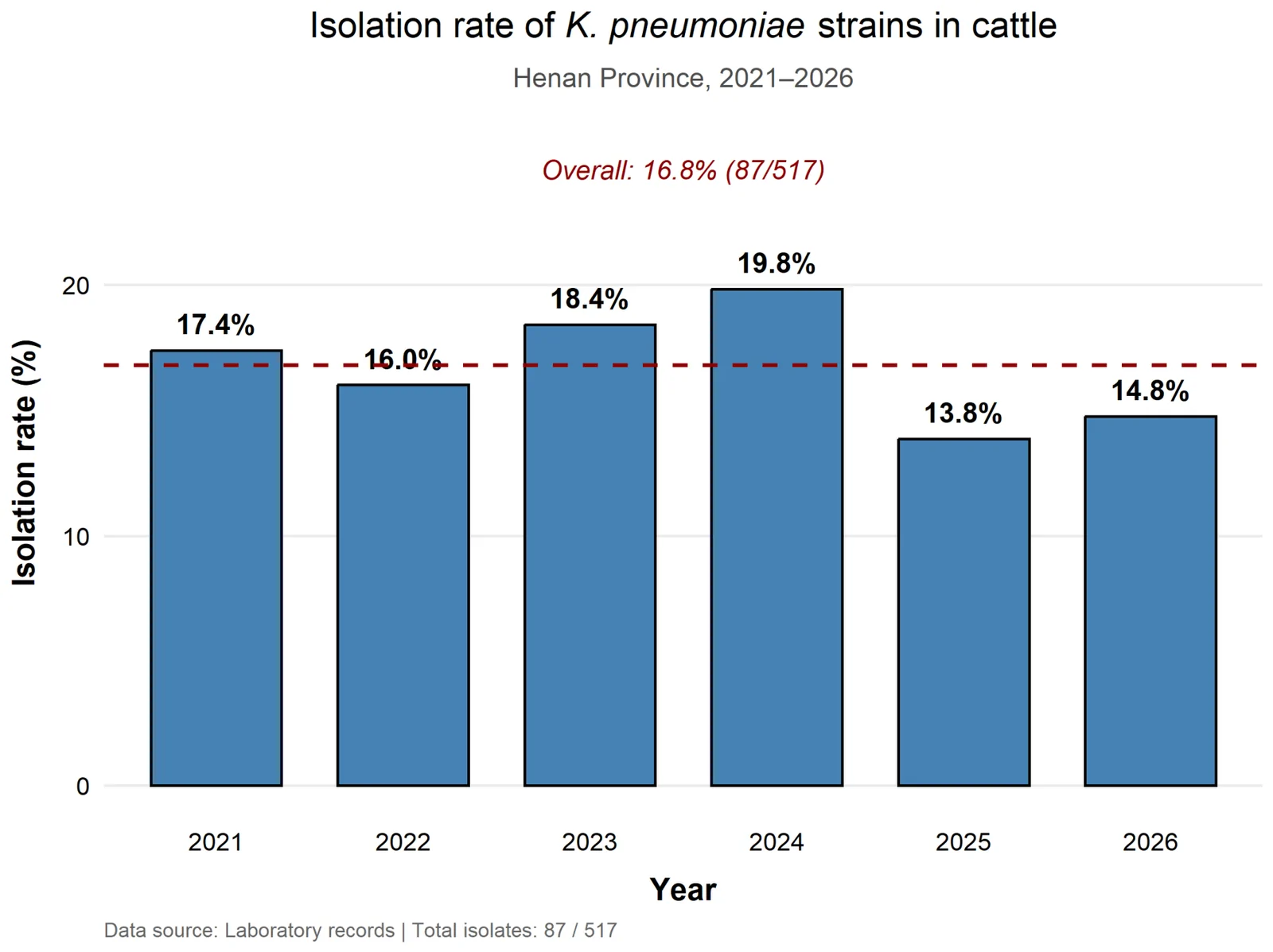
Isolation rate of *K. pneumoniae* strains in cattle from 2021 to 2026 in Henan province.

In this study, 87 out of 517 deep nasal swab samples yielded bacterial isolates that were confirmed as *K. pneumoniae* through Gram staining and species-specific PCR, resulting in an overall isolation rate of 16.8%. The annual sample sizes and positive counts are presented in Figure 1 and Table S1. Briefly, the numbers of samples collected per year were 46 (2021), 50 (2022), 114 (2023), 116 (2024), 130 (2025), and 61 (2026). The overall isolation rate was 16.8% (87/517; 95% CI 13.9–20.3). Annual isolation rates with 95% confidence intervals were 17.4% (9.1–30.7) in 2021, 16.0% (8.3–28.5) in 2022, 18.4% (12.4–26.5) in 2023, 19.8% (13.6–28.0) in 2024, 13.8% (8.9–20.8) in 2025, and 14.8% (8.0–25.7) in 2026. (Figure 1). Geographically, isolation rates varied across five regions of Henan province, with east Henan showing the numerically highest rate (22.6%, 14/62; 95% CI: 13.8–35.5), followed by west (20.8%, 11/53; 95% CI: 11.9–34.4), central (16.8%, 16/95; 95% CI: 10.5–26.1), south (15.5%, 34/220; 95% CI: 11.2–20.9), and north (13.8%, 12/87; 95% CI: 8.0–22.7). However, the 95% confidence intervals for all regions overlapped substantially, and the regional differences were not statistically significant after accounting for farm-level clustering in a mixed-effects logistic regression model (farm random intercept variance = 0.42; ICC = 0.11; likelihood ratio test for the random effect: *p* = 0.18). These regional estimates had unequal denominators and overlapping 95% confidence intervals after accounting for farm clustering. A Pearson chi-square test for heterogeneity yielded χ^2^ = 1.78, df = 5, *p* = 0.879, indicating no statistically significant difference in isolation rates among the six years (2021–2026). This test assessed heterogeneity across years and was not a trend test. All Bonferroni-adjusted *p*-values were ≥0.99, confirming no significant pairwise differences between any two years. At the city level, the numerically highest and lowest isolation rates were observed in Shangqiu (25.0%, 3/12; 95% CI: 8.7–53.1) and Puyang (9.1%, 1/11; 95% CI: 1.6–37.7), respectively; however, these estimates were based on very small denominators and the confidence intervals were extremely wide, precluding any meaningful comparison. A summary of all isolates is provided in Table 1 and Table S1.

### 3.2. MLST Sequence Type Distribution

**Figure 2:**
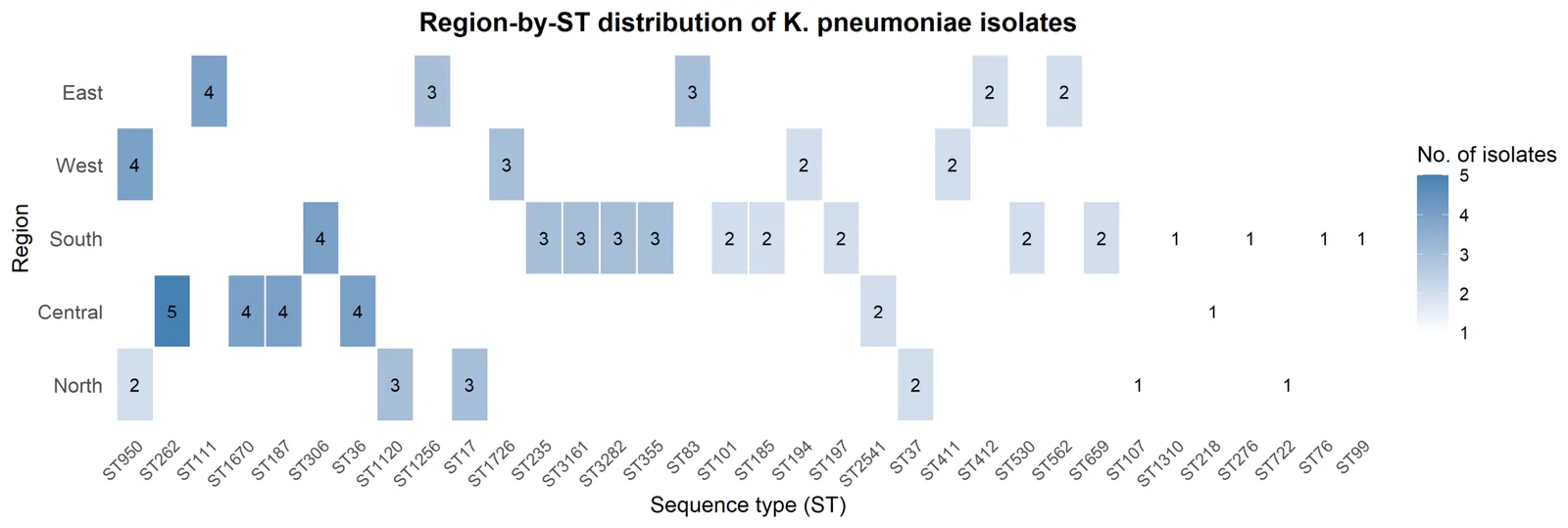
Region-by-ST distribution of 87 *K. pneumoniae* isolates from calves with BRD in five regions of Henan Province, China.

A total of 87 *K. pneumoniae* isolates recovered from nasal swabs of cattle with bovine respiratory disease (BRD) across five regions of Henan province (North, Central, South, West, East) were characterized by multilocus sequence typing (MLST). The region-by-ST heatmap (Figure 2) shows the distribution of each ST across the five geographic regions. The analysis revealed 34 distinct STs, indicating considerable genetic diversity (Figure 2). The most prevalent ST was ST950, identified in northern (n = 2) and western (n = 4) regions, accounting for six isolates (6.9% of typed strains). ST262 (n = 5) and ST187 (n = 4) were exclusively found in the central region, while ST306 (n = 4) was restricted to the south. ST111 (n = 4) and ST412 (n = 2) were observed only in the east, and ST1726 (n = 3) only in the west. Several other STs, including ST1670, ST36, ST2541, and ST3282, were present in low frequencies (1–4 isolates each), with distinct geographical clustering. These findings indicate a heterogeneous distribution of *K. pneumoniae* STs across Henan, with certain STs showing regional clustering. Whether this reflects local transmission dynamics or host adaptation remains a hypothesis because MLST has limited discriminatory resolution and no phylogenomic or epidemiological linkage analysis was performed.

The heatmap shows the number of isolates for each sequence type (ST) across five geographic regions (North, Central, South, West, East). STs are ordered by total isolate count (descending). Cells are coloured according to the number of isolates, with the exact count shown in each cell.

### 3.3. Antimicrobial Susceptibility

**Figure 3:**
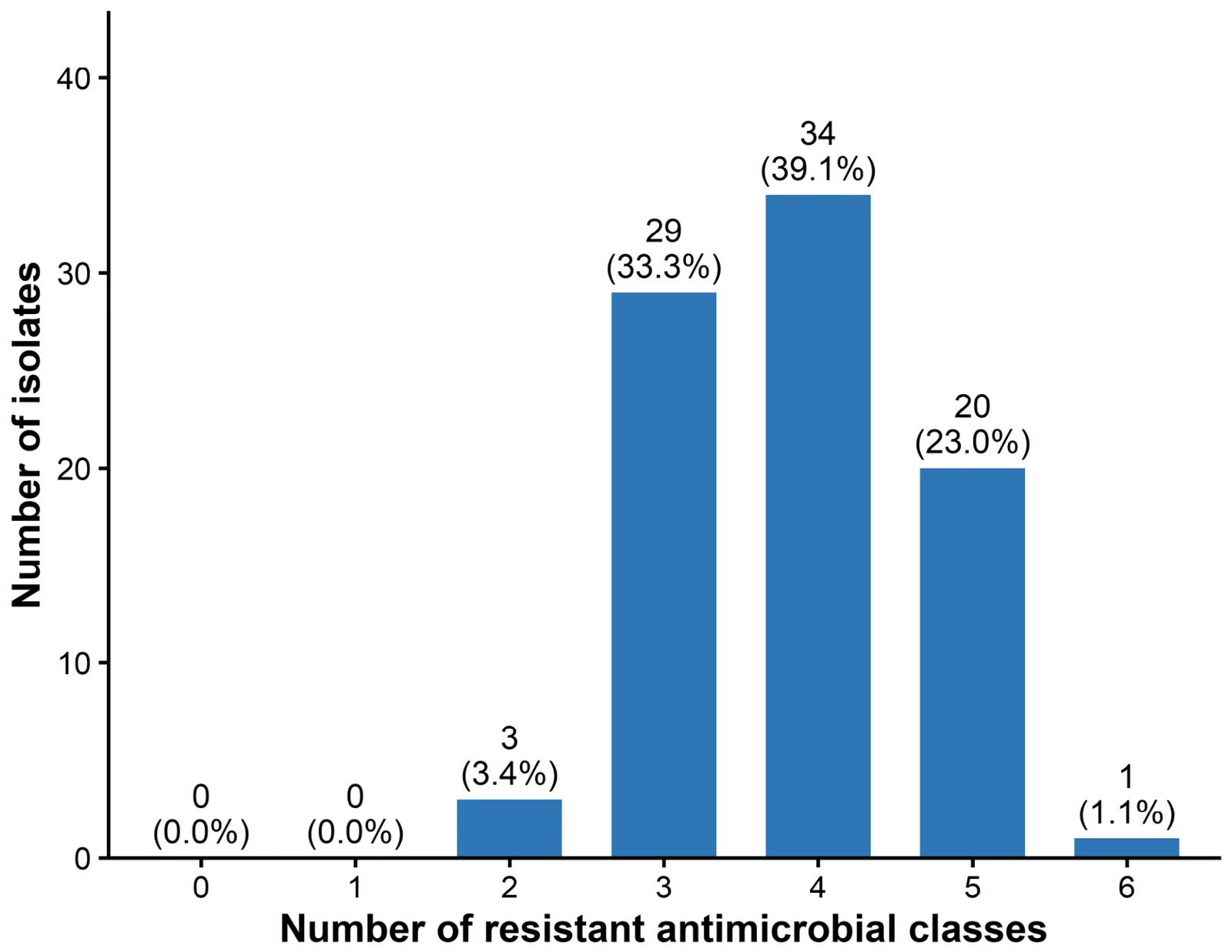
Distribution of the number of antimicrobial classes to which the 87 *K. pneumoniae* isolates were resistant. The bar chart shows the number of isolates (y-axis) resistant to 0, 1, 2, 3, 4, 5, or 6 antimicrobial classes (x-axis).

The 87 *K. pneumoniae* strains were classified as susceptible, intermediate, or resistant to 15 antibiotics across six classes. As presented in Table 2, isolates from different regions exhibited varying resistance profiles. The antibiotic resistance rates for agents with CLSI interpretive criteria, in descending order, were: ampicillin (94.3%), sulfisoxazole (79.3%), cefoxitin (73.6%), cefazolin (70.1%), enrofloxacin (67.8%), cefixime (62.1%), levofloxacin (59.8%), amoxicillin-clavulanate (59.8%), tetracycline (55.2%), trimethoprim-sulfamethoxazole (46.0%), kanamycin (40.2%), amikacin (36.8%), doxycycline (34.5%), gentamicin (25.3%), and florfenicol (11.5%). MDR was defined as resistance to at least one agent in ≥3 of the six classes: β-lactams, sulfonamides, tetracyclines, phenicols, aminoglycosides, and quinolones. Intermediate isolates were classified as non-resistant. Among the 87 isolates, 96.6% (84/87; 95% CI 90.3–98.8%) were MDR. The distribution of the number of resistant classes is shown in Figure 3. Specifically, 3 isolates (3.4%) were resistant to 2 classes, 29 (33.3%) to 3 classes, 34 (39.1%) to 4 classes, 20 (23.0%) to 5 classes, and 1 (1.1%) to 6 classes; no isolate was susceptible to all classes or resistant to only 1 class.

For geographical analysis, isolate-level class resistance—defined as resistance to at least one agent within a class—was summarized for the five regions (Table 3). β-lactam resistance ranged from 92.9% to 100.0%, sulfonamide resistance from 87.5% to 92.9%, tetracycline resistance from 54.5% to 64.3%, phenicol resistance from 8.3% to 14.3%, aminoglycoside resistance from 50.0% to 54.5%, and quinolone resistance from 72.7% to 78.6%. Overall resistance was highest for β-lactams (98.9%) and sulfonamides (89.7%), followed by quinolones (74.7%), tetracyclines (58.6%), aminoglycosides (51.7%), and phenicols (11.5%). The numerically lower resistance proportions observed in North Henan for some classes should be interpreted as descriptive only; no formal statistical comparison was performed because of unequal sample sizes across regions and farm-level clustering.

### 3.4. Detection of Antimicrobial Resistance Genes

**Figure 4:**
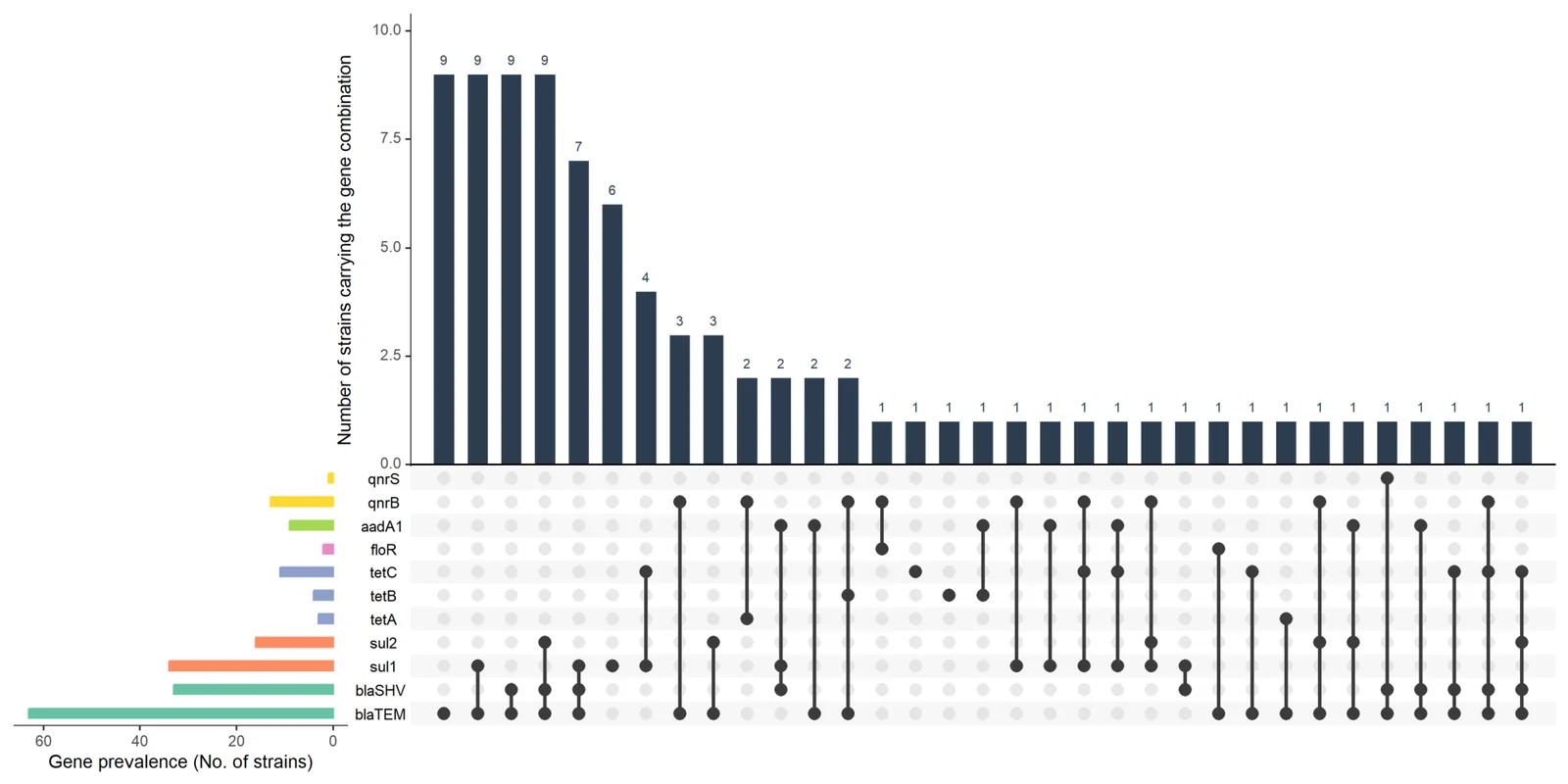
The distribution of antibiotic resistance genes among 87 *K. pneumoniae* strains.

A total of 22 antimicrobial resistance genes, representing six distinct drug classes, were screened via PCR, leading to the identification of 11 genes across these classes. Regarding β-lactam resistance, *blaTEM* was the most prevalent gene (72.4%), followed by *blaSHV* (37.9%). Notably, *blaVIM* and *blaCTX-M-2* were not detected. For sulfonamide resistance, *sul1* (39.1%) was more common than *sul2* (18.4%). Among tetracycline resistance genes, *tetC* (12.6%) was detected more frequently than *tetB* (4.6%) and *tetA* (3.4%). The only phenicol resistance gene identified was *floR* (2.3%). The aminoglycoside resistance gene (*aadA1*) was found in 10.3% of isolates. Among quinolone resistance determinants, *qnrB* and *qnrS* were detected at rates of 14.9% and 1.1%, respectively, while other resistance genes were not found (Figure 4).

As detailed in Figure 4 and Table S4, 17 strains harbored only a single resistance gene, while the remaining 70 carried two or more. The most prevalent multi-gene patterns were *blaTEM* + *blaSHV*, *blaTEM* + *blaSHV* + *sul2*, and *blaTEM* + *sul1*, each occurring in 10.34% of strains. This was followed by the *blaTEM* + *blaSHV* + *sul1* pattern (8.05%), and then *blaTEM* + *sul2* and *blaTEM* + *qnrB* (both 3.45%). Four other patterns (*aadA1* + *blaSHV* + *sul1*, *qnrB* + *tetA*, *aadA1* + *blaTEM*, and *blaTEM* + *qnrB* + *tetB*) were each found in two strains (2.3%). The remaining strains exhibited unique patterns.

Associations between resistance genes and phenotypic resistance were assessed at the antimicrobial class level using 2 × 2 contingency tables (Table 4). After FDR correction, no gene was significantly associated with phenotypic resistance (all q > 0.05). The strongest nominal association was observed between sul1 and sulfonamide resistance (OR = 4.17, 95% CI 0.89–19.61; cluster-adjusted *p* = 0.070; q = 0.422), which did not remain significant after FDR correction. For gene–class pairs with one-sided complete separation or sparse data (*blaSHV, blaTEM, tetA, floR,* and *qnrS*), ORs and cluster-adjusted *p*-values could not be estimated and are reported as NE. No other gene–class pair reached statistical significance.

### 3.5. Detection of Virulence-Associated Genes

**Figure 5:**
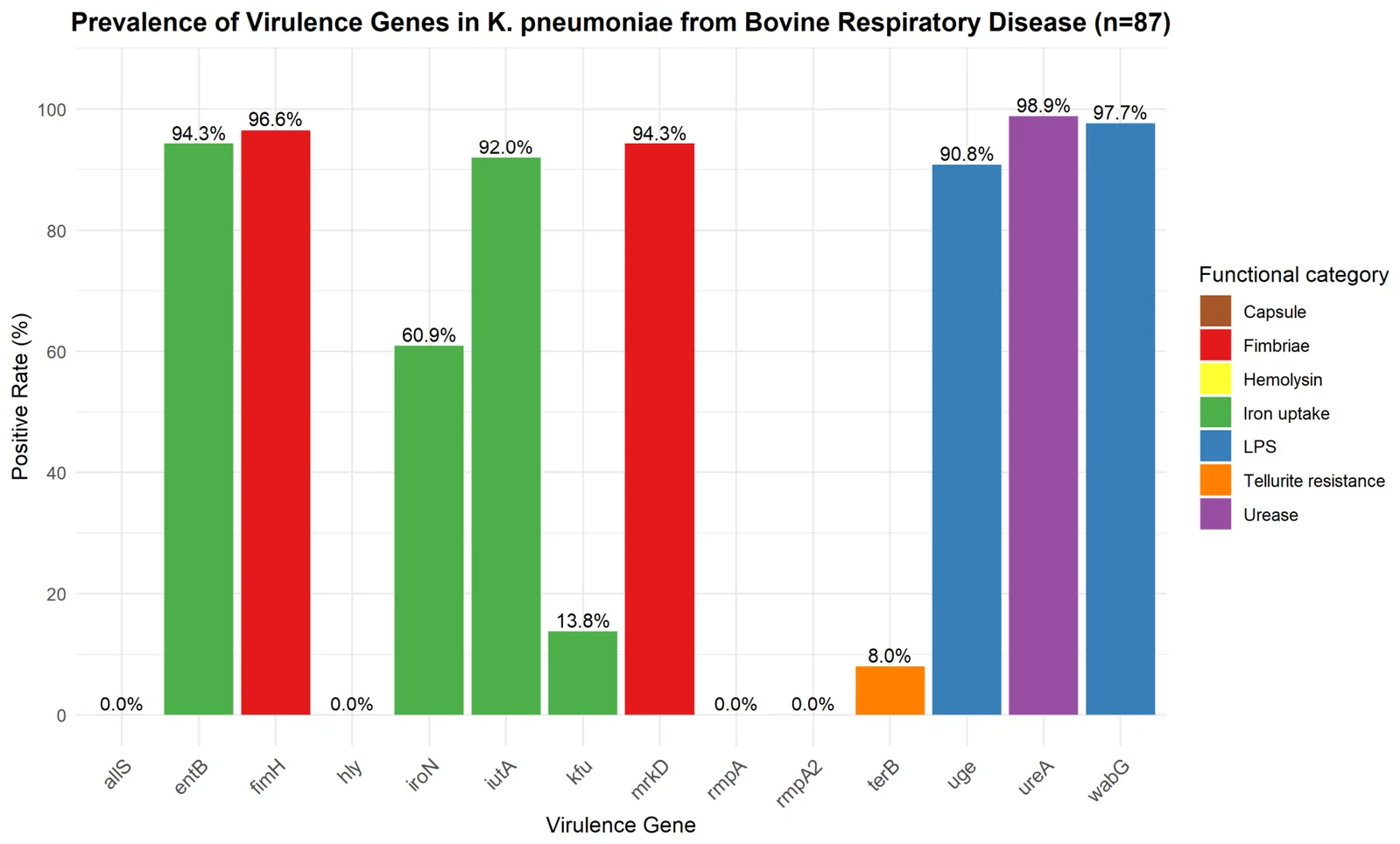
The distribution of virulence-associated genes among 87 *K. pneumoniae* strains.

The detailed status of virulence-associated genes carried by 87 *K. pneumoniae* strains is shown in Figure 5. Among the fimbriae synthesis-related genes, *fimH* and *mrkD* were detected in 84 (96.6%) and 82 (94.3%) strains, respectively. For lipopolysaccharide-related genes, *uge* was present in 79 strains (90.8%), while *wabG* was found in 85 strains (97.7%). Regarding iron uptake system genes, *entB* and *iutA* were identified in 82 (94.3%) and 80 (92.0%) strains, respectively; *iroN* was positive in 53 strains (60.9%), and *kfu* was present in only 12 strains (13.8%). The urease-related gene *ureA* was detected in 86 strains (98.9%), whereas *allS* was not detected in any strain (0%). The tellurite resistance gene *terB* was found in 8 strains (9.2%). Notably, the hemolysin gene (*hly*), the capsular polysaccharide synthesis gene and the regulation-related genes (*rmpA* and *rmpA2*) were absent in all 87 isolates (0%). Because positive controls confirmed assay functionality, these genes were not detected in the tested isolates; however, this does not exclude the presence of other hypervirulence-associated determinants. PCR detection indicates carriage only and does not demonstrate expression or functional virulence.

According to the statistics of multiple virulence-associated gene carriers, all 87 isolates carried at least six virulence-associated genes. The most prevalent combination was *fimH* + *mrkD* + *uge* + *wabG* + *entB* + *iutA* + *iroN* + *ureA* (eight genes), which was observed in 31 isolates (35.6%). The second most common profile was *fimH* + *mrkD* + *uge* + *wabG* + *entB* + *iutA* + *ureA* (seven genes, lacking *iroN*), present in 24 isolates (27.6%). A smaller proportion of isolates (9.2%, 8/87) carried the eight-gene combination together with *terB*. The least frequent profiles among those with at least six genes were unique patterns carried by single isolates (e.g., ST99 with *fimH*-negative but positive for *mrkD*, *uge*, *wabG*, *entB*, *iutA*, *iroN*), each accounting for only 1.1% (1/87). These results indicate that multiple virulence-associated gene carriage is common in bovine respiratory *K. pneumoniae* isolates from Henan, with a dominant profile comprising fimbrial, lipopolysaccharide, iron uptake, and urease genes.

## 4. Discussion

In this study, the isolation rate of *K. pneumoniae* was 16.8%. This rate is higher than reports from dairy cows with BRD in Hebei (37/316, 11.7%) [6] and cattle with BRD in Chongqing (33/213, 15.5%) [19] in China, but lower than the isolation rate from pneumonic lung tissues, and tracheal swabs were collected from Aceh cattle in Indonesia (38/61,62.3%) [14]. These comparisons should be interpreted with caution because nasal swabs from live BRD calves cannot be directly compared with pneumonic lung tissue or tracheal samples without considering differences in diagnostic sensitivity, colonization, disease severity, and sampling context. *K. pneumoniae* is an opportunistic pathogen that can colonize the upper respiratory tract of healthy cattle. Because only nasal swabs were collected, this study cannot distinguish colonization from infection or determine whether *K. pneumoniae* was the sole causative agent of BRD. The Wisconsin Calf Respiratory Scoring System is a field screening tool, not a diagnostic gold standard. Coinfection with *Mannheimia haemolytica*, *Pasteurella multocida*, *Mycoplasma bovis*, or other BRD pathogens was not assessed. Therefore, the role of *K. pneumoniae* as a sole or contributory pathogen could not be determined by this method. Geographically, the isolation rates were numerically lower in North (13.8%) and South (15.5%) Henan than in East (22.6%) and West (20.8%) Henan. However, the 95% confidence intervals for all regional estimates overlapped substantially, and after accounting for farm-level clustering in a mixed-effects logistic regression model, no regional difference was statistically significant. The intraclass correlation coefficient was 0.11, indicating that a modest proportion of the total variation in isolation status was attributable to differences between farms. This clustering is biologically plausible, as calves within the same farm share management practices, housing conditions, and antimicrobial exposure histories. The regional comparisons in this study should therefore be interpreted with caution because the sample sizes varied considerably across regions (range: 53–220 samples) and the study was not powered to detect small regional differences. The absence of statistically significant regional variation does not necessarily indicate that true regional differences are absent; rather, it may reflect limited statistical power. Future studies with balanced sampling across regions and larger sample sizes are needed to rigorously evaluate geographic patterns. Although isolation rates fluctuated numerically across years, no statistically significant temporal trend was detected (Pearson chi-square test for heterogeneity: χ^2^ = 1.78, df = 5, *p* = 0.879). The isolation rate remained above 10.0% in all years, indicating that *K. pneumoniae* was consistently present in calves with BRD in Henan Province. However, because no trend test was performed and no significant annual difference was found, the data should not be interpreted as evidence of a declining or increasing temporal trend.

In this study, we isolated 87 *K. pneumoniae* strains from cattle with pneumonia, which were classified into 34 STs. ST950 was the most frequently identified ST; ST950 has been associated with hypervirulent *K. pneumoniae* (hvKP) in previous human studies [56]. However, ST designation alone is insufficient to infer hypervirulence for these bovine isolates, and hypervirulence markers were absent in our collection. Other notable STs included ST262 (5.7%, 5/87), ST306 (4.6%, 4/87), ST187 (4.6%, 4/87), and others. These STs have also been reported in other studies at low detection rates [6,20]. Notably, ST262 was previously isolated and reported from sheep in Henan Province [20]. Given that some of the cattle farms in this study were backyard farms where cattle and sheep shared grazing land, the shared ST262 between cattle and sheep suggests a hypothesis of possible cross-species transmission; however, MLST has limited discriminatory power, and whole-genome sequencing with epidemiological linkage is required to test this hypothesis. Moreover, isolates from cattle exhibited a high diversity of STs. A survey identified 19 STs among 37 *K. pneumoniae* strains from cattle with BRD, with ST43 as the predominant type (29.7%) in Hebei province, China [6]. In Chongqing city, 7 STs were identified among 33 *K. pneumoniae* strains from cattle with BRD, with ST218 being the most common (57.6%) [19]. *K. pneumoniae* can infect not only cattle, causing pneumonia, but also the mammary glands of dairy cows, leading to mastitis. Moreover, the STs of mastitis-associated strains also display diversity. One study across seven provinces found 42 STs among 108 *K. pneumoniae* isolates, with ST1049 as the major type (11.1%) [17]. Additionally, an investigation in Hubei province detected 100 STs among 239 *K. pneumoniae* isolates, with ST2854 as the dominant type (12.6%) [28]. However, among these three predominant types found in these studies, only ST218 was detected in our study, at a rate of 1.1%. In USA, 23 STs were detected in 29 *K. pneumoniae* strains, including ST101 (13.0%), ST114 (13.0%), and ST112 (13.0%) [57]; only ST101 was observed in the present study. Comparing these findings, integrated surveillance systems are needed for monitoring *K. pneumoniae* across animal, human, and environmental compartments.

In China, antibiotics remain the primary approach for treating bacteria-associated BRD. However, recent reports have shown an increasing trend of bacterial resistance to antimicrobial agents among *K. pneumoniae* isolates [6,19]. Antimicrobial susceptibility testing is essential to guide the selection of appropriate drugs for infection treatment. In this study, the highest resistance was observed against β-lactams, which is consistent with findings from previous reports on *K. pneumoniae* isolates from dairy cows with BRD [6] or with mastitis in China [28]. Additionally, sulfonamides resistance ranged from 87.5% to 92.9% across regions, while resistance to quinolones ranged from 72.7% to 78.6% across regions. These rates are comparable to or exceed those reported for bovine *K. pneumoniae* in China [6,17]. This trend is not surprising, as sulfonamides and quinolones are among the most frequently used antibiotics in dairy animals in China [58]. The high resistance rates may be associated with antimicrobial selection pressure; however, quantitative antimicrobial-use data were not collected, and this mechanism cannot be established from the present data. Their extensive use likely contributes to the rising resistance in *K. pneumoniae*. Moreover, the resistance rates of the isolates to the remaining three antimicrobial classes were lower than those for the three classes mentioned above, with phenicols showing the lowest resistance rate. These results highlight the urgent need for prudent antimicrobial use in treating cattle *K. pneumoniae*. The challenges of antimicrobial management in BRD are further illustrated by Masebo et al. (2024), who investigated BRD in fattening bulls under commercial conditions and reported a high disease burden, extensive antimicrobial treatment, and persistent pulmonary lesions at slaughter [9]. These observations reinforce the need for antimicrobial stewardship and susceptibility-guided treatment in cattle production. Therefore, the clinical use of β-lactams, sulfonamides, and quinolones should be minimized in Henan province unless supported by susceptibility testing. Florfenicol showed the lowest resistance rate among the tested agents. However, in vitro susceptibility alone cannot establish clinical preference, and treatment decisions should be guided by susceptibility testing, clinical outcome data, and antimicrobial stewardship.

β-lactamase-mediated hydrolysis is a primary mechanism of β-lactam resistance, thereby rendering these drugs ineffective [59]. In this study, *blaTEM* was detected in 72.4% of *K. pneumoniae* isolates, lower than the rates anged from 87.5% to 92.9% reported (92.6%) in China [28] and in South Africa (85.5%) [60]. Sulfonamide resistance mediated by the *sul* gene has become widespread globally, particularly in *K. pneumoniae* from dairy cattle with mastitis, where resistance to sulfonamide antibiotics is pronounced [61]. In this study, the detection rates of *sul1* and *sul2* were 39.1% and 18.4%, respectively, consistent with previously reported data on *K. pneumoniae* isolated from mastitis cases in China [17]. Generally, *tetA*, *tetB*, and *tetC* are the most common tetracycline resistance genes in *K. pneumoniae* of animal origin, and these genes are part of the small nonconjugative transposons in plasmids [62]. Among the isolates in this study, the detection rates of these three genes were 3.4%, 4.6%, and 12.6%, respectively, which were higher than the rates in earlier studies in dairy cattle [28]. The only phenicol resistance gene identified was *floR* (2.3%), lower than the rates in cattle in China [28] and Egypt [63]. Resistance to aminoglycosides is conferred by genes encoding aminoglycoside-modifying enzymes, such as the *aadA1* gene [64]. Quinolone resistance is associated with plasmid-mediated quinolone resistance genes, such as *qnrB* and *qnrS* genes [15,65]. As in this study, these resistance genes have also been detected in isolates from cows in China with varying prevalence rates [6,17,44]. After FDR correction for multiple comparisons, no gene was significantly associated with phenotypic resistance. hese results show profiles different from those in previous work, where correlations of some genes have been observed in *K. pneumoniae* isolates from dairy cow, chicken, sheep, and pig in Xinjiang in China [66]. The wide confidence intervals and lack of significant associations likely reflect the limited number of isolates (n = 87), the class-level resistance definition, the presence of other resistance mechanisms not captured by the PCR panel, and farm-level clustering. These findings highlight the complexity of genotype–phenotype relationships and the need for whole-genome sequencing to fully characterize resistance mechanisms.

The pathogenicity of *K. pneumoniae* is critically dependent on virulence factors. In the present study, genes linked to hypervirulence—*rmpA*, *rmpA2*, and *hly*—were not detected. However, absence of the screened markers alone cannot exclude hypervirulence, and carriage of several common virulence-associated genes cannot establish pathogenic potential. Functional assays, capsule typing, siderophore quantification, biofilm assays, infection models, or genomic characterization would be needed for stronger conclusions. Additionally, PCR analysis revealed that fimbria-associated genes (*fimH*, *mrkD*), iron-acquisition system genes (*iutA*, *iroN*, *entB*), urease gene (*ureA*), and lipopolysaccharide-related genes (*uge*, *wabG*) were broadly distributed among the isolates, consistent with previous reports which have also been reported in isolates from cows in many provinces in China [17,28]. These findings offer preliminary elucidation of the pathogenic mechanisms of *K. pneumoniae*, which may involve: assembling fimbriae to attach to host cell surfaces or to form biofilms that promote virulence; secreting siderophores to capture host iron for metabolism and enhanced virulence; employing capsular polysaccharides to evade serum-mediated killing by phagocytes and to dampen host immunity; and organizing lipopolysaccharide into complexes on the bacterial surface, enabling the pathogen to escape or resist killing by the host’s innate immune system [67,68]. In addition, Wan et al. investigated similar virulence-associated genes in *K. pneumoniae* strains obtained from pigs in Northwest China [25]. Another study by Hou et al. (2024) also identified comparable virulence-associated genes in *K. pneumoniae* isolated from dairy cows, chicken, sheep, and pigs in Xinjiang, China [66]. These studies suggest that virulence-associated genes in *K. pneumoniae* may exhibit similarities and broad distribution among strains from different host origins. Whether this is related to the transmission of *K. pneumoniae* across different hosts remains unclear and warrants further investigation.

Future whole-genome sequencing studies are warranted to further elucidate resistance mechanisms, transmission pathways, and host-adaptation signatures of this pathogen in cattle. Longitudinal surveillance is also valuable in intensive cattle production. Dini et al. (2024) showed in a longitudinal study of fattening beef cattle that infectious exposure can change over time in relation to farm management and biosecurity conditions [10]. Although that study concerned *Toxoplasma gondii* rather than *K. pneumoniae* or BRD, it illustrates the broader value of longitudinal monitoring for understanding infectious disease dynamics in intensively managed cattle.

This study has several important limitations. First, no clinically healthy control calves were included; therefore, the isolation rate among BRD-positive calves cannot establish an association between *K. pneumoniae* carriage and BRD. Second, only deep nasal swabs were collected, and no lung histopathology, pathogen load quantification, or challenge–re-isolation experiments were performed; thus, the study cannot distinguish colonization from infection or determine whether *K. pneumoniae* was the sole causative agent. Third, coinfection with other core BRD pathogens such as *Mannheimia haemolytica*, *Pasteurella multocida*, and *Mycoplasma bovis* was not assessed. Fourth, only one confirmed *K. pneumoniae* isolate per nasal swab was stored and characterized. Because a single sample may contain multiple strains with different STs, this approach may underestimate within-host strain diversity and population heterogeneity. Fifth, farm-level clustering was present and was accounted for where possible, but some analyses remain descriptive.

## 5. Conclusions

This study provides the first comprehensive molecular and phenotypic characterization of *K. pneumoniae* isolates from cattle with BRD in Henan Province, China, covering a six-year period (2021–2026). *K. pneumoniae* was isolated from 16.8% of sampled calves with BRD. Because no healthy comparison group or etiological confirmation was included, this study cannot establish *K. pneumoniae* as a causative agent of BRD; the organism may be a colonizer, an opportunistic pathogen, or a component of a polymicrobial infection. The 87 isolates were assigned to 34 diverse STs, indicating substantial genetic heterogeneity. The shared ST262 with sheep suggests a hypothesis of possible cross-species transmission that requires whole-genome sequencing and epidemiological linkage for confirmation. Notably, 96.6% of isolates were multidrug-resistant, with extremely high resistance to β-lactams, sulfonamides, and quinolones. After FDR correction, no resistance gene was significantly associated with phenotypic resistance. Multiple virulence-associated gene carriage is common in bovine respiratory *K. pneumoniae* isolates from Henan, with a dominant profile comprising fimbrial, lipopolysaccharide, iron uptake, and urease genes. However, carriage of these genes does not establish pathogenic potential. The dominant virulence-associated gene profile (*fimH* + *mrkD* + *uge* + *wabG* + *entB* + *iutA* + *iroN* + *ureA*) was found in over one-third of isolates, but gene prevalence alone does not establish expression, immunogenicity, essentiality, or protective potential, and these findings should not be presented as a basis for vaccine or therapeutic target development without further functional studies. Antimicrobial stewardship, susceptibility-guided treatment, and whole-genome sequencing are warranted to resolve *K. pneumoniae* strain relatedness, characterize resistance determinants, investigate mobile genetic elements, and provide stronger evidence regarding possible transmission between farms, regions, and animal species.

## Figures and Tables

**Table 1:** Nasal swab samples collected from calves with BRD on cattle farms in five regions of Henan province from 2021 to 2026.

Region	City	Farm	Breed	Samples, n	Samples with Confirmed *K. pneumoniae*	
					By Herd, n (%)	By Region,%
North	Anyang	1	Simmental	12	2 (16.7)	12/87 (13.8)
		2	Angus	15	2 (13.3)	
	Hebi	3	Simmental	10	1 (10.0)	
		4	Simmental	17	3 (17.6)	
	Puyang	5	Simmental	11	1 (9.1)	
	Xinxiang	6	Charolais	14	2 (14.3)	
		7	Simmental	8	1 (12.5)	
Central	Pingdingshan	8	Simmental	16	4 (25.0)	16/95 (16.8)
		9	Charolais	15	1 (6.7)	
		10	Simmental	20	4 (20.0)	
		11	Simmental	18	4 (22.2)	
	Luohe	12	Xianan	14	2 (14.3)	
		13	Simmental	12	1 (8.3)	
South	Nanyang	14	Simmental	15	2 (13.3)	34/220 (15.5)
		15	Simmental	19	4 (21.1)	
		16	Simmental	13	3 (23.1)	
		17	Nanyang	9	2 (22.2)	
		18	Simmental	12	2 (16.7)	
		19	Xianan	21	4 (19.0)	
		20	Nanyang	13	2 (15.4)	
		21	Nanyang	8	1 (12.5)	
		22	Xianan	14	1 (7.1)	
		23	Nanyang	14	3 (21.4)	
		24	Simmental	10	1 (10.0)	
	Xinyang	25	Simmental	12	1 (8.3)	
		26	Nanyang	11	1 (9.1)	
		27	Simmental	20	2 (10.0)	
	Zhumadian	28	Simmental	13	2 (15.4)	
		29	Nanyang	16	3 (18.8)	
West	Luoyang	30	Xianan	12	2 (16.7)	11/53 (20.8)
		31	Simmental	17	4 (23.5)	
	Sanmenxia	32	Simmental	9	2 (22.2)	
		33	Xianan	15	3 (20.0)	
East	Shangqiu	34	Simmental	11	3 (27.3)	14/62 (22.6)
		35	Charolais	10	2 (10.0)	
		36	Simmental	15	4 (26.7)	
	Zhoukou	37	Simmental	14	3 (21.4)	
		38	Simmental	12	2 (16.7)	
Total				517		87/517 (16.8)

**Table 2:** Antimicrobial agents, disk contents, interpretive criteria, sources, and resistance profiles of 87 *K. pneumoniae* isolates.

Antimicrobial Class	Antimicrobial Agent	Disk Content (μg/Piece)	Zone Diameter Breakpoints (mm), S/I/R	Source	No. (%) Susceptible	No. (%) Intermediate	No. (%) Resistant
β-lactams	Ampicillin	10	≥17/14–16/≤13	CLSI VET01S	5 (5.7)	0 (0.0)	82 (94.3)
	Amoxicillin-clavulanate	20/10	≥18/14–17/≤13	CLSI M100-S26	26 (29.9)	9 (10.3)	52 (59.8)
	Cefazolin	30	≥23/20–22/≤19	CLSI M100-S26	22 (25.3)	4 (4.6)	61 (70.1)
	Cefoxitin	30	≥18/15–17/≤14	CLSI M100-S26	20 (23.0)	3 (3.4)	64 (73.6)
	Cefixime	5	≥19/16–18/≤15	CLSI M100-S26	30(34.5)	3(3.4)	54(62.1)
Sulfonamides	Sulfisoxazole	300	≥17/13–14/≤12	CLSI VET01S	18(20.7)	0(0.0)	69(79.3)
	Trimethoprim-sulfamethoxazole	1.25/23.75	≥16/11–15/≤10	CLSI VET01S	45 (51.7)	2 (2.3)	40 (46.0)
Tetracyclines	Tetracycline	30	≥15/12–14/≤11	CLSI VET01S	35 (40.2)	4 (4.6)	48 (55.2)
	Doxycycline	30	≥14/11–13/≤10	CLSI VET01S	52 (59.8)	5 (5.7)	30 (34.5)
Phenicols	Florfenicol	30	≥18/13–17/≤12	CLSI VET01S	73 (83.9)	4 (4.6)	10 (11.5)
Aminoglycosides	Gentamicin	10	≥15/13–14/≤12	CLSI VET01S	65 (74.7)	0 (0.0)	22 (25.3)
	Amikacin	30	≥17/15–16/≤14	CLSI VET01S	55 (63.2)	0 (0.0)	32 (36.8)
	Kanamycin	30	≥18/14–17/≤13	CLSI M100-S26	50 (57.5)	2 (2.3)	35 (40.2)
Quinolones	Levofloxacin	5	≥17/14–16/≤13	CLSI M100-S26	35 (40.2)	0 (0.0)	52 (59.8)
	Enrofloxacin	5	≥15/11–14/≤10	CLSI VET01S	28 (32.2)	0 (0.0)	59 (67.8)

Note: S = susceptible; I = intermediate; R = resistant. CLSI breakpoints were from CLSI VET01S and CLSI M100-S26.

**Table 3:** Isolate-level class resistance of *K. pneumoniae* isolates from five regions of Henan Province, China.

Region	No. of Isolates	β-Lactams n (%)	Sulfonamides n (%)	Tetracyclines n (%)	Phenicols n (%)	Aminoglycosides n (%)	Quinolones n (%)
North	12	12 (100.0)	11 (91.7)	7 (58.3)	1 (8.3)	6 (50.0)	9 (75.0)
Central	16	16 (100.0)	14 (87.5)	9 (56.3)	2 (12.5)	8 (50.0)	12 (75.0)
South	34	34 (100.0)	30 (88.2)	20 (58.8)	4 (11.8)	18 (52.9)	25 (73.5)
West	11	11 (100.0)	10 (90.9)	6 (54.5)	1 (9.1)	6 (54.5)	8 (72.7)
East	14	13 (92.9)	13 (92.9)	9 (64.3)	2 (14.3)	7 (50.0)	11 (78.6)
Overall	87	86 (98.9)	78 (89.7)	51 (58.6)	10 (11.5)	45 (51.7)	65 (74.7)

Note: Isolate-level class resistance was defined as resistance to at least one agent within a class.

**Table 4:** Genotype–phenotype associations for antimicrobial resistance in *K. pneumoniae* isolates.

Antimicrobial Class	Gene	Gene+/R (a)	Gene+/S/I (b)	Gene−/R (c)	Gene−/S/I (d)	OR (95% CI)	Cluster-Adjusted *p*	FDR q
β-Lactams	*blaSHV*	33	0	53	1	NE	NE	NE
β-Lactams	*blaTEM*	63	0	23	1	NE	NE	NE
Sulfonamides	*sul1*	33	1	45	8	4.17 (0.89–19.61)	0.070	0.422
Sulfonamides	*sul2*	13	3	65	6	0.38 (0.08–1.79)	0.222	0.631
Tetracyclines	*tetA*	3	0	48	36	NE	NE	NE
Tetracyclines	*tetB*	3	1	48	35	1.71 (0.33–8.93)	0.526	0.631
Tetracyclines	*tetC*	7	4	44	32	1.22 (0.42–3.52)	0.717	0.717
Phenicols	*floR*	0	2	10	75	NE	NE	NE
Aminoglycosides	*aadA1*	6	3	39	39	1.86 (0.52–6.64)	0.341	0.631
Quinolones	*qnrB*	11	2	54	20	1.73 (0.38–7.83)	0.476	0.631
Quinolones	*qnrS*	0	1	65	21	NE	NE	NE

Note: (a) = gene-positive and resistant to ≥1 agent in the class; (b) = gene-positive and susceptible/intermediate to all agents in the class; (c) = gene-negative and resistant to ≥1 agent in the class; (d) = gene-negative and susceptible/intermediate to all agents in the class. OR (95% CI) was obtained from Firth’s penalized logistic regression with farm-level cluster-robust standard errors. NE = not estimable due to complete or quasi-complete separation or sparse data. FDR q-values were calculated using the Benjamini–Hochberg procedure based on cluster-adjusted *p*-values.

## Data Availability

The original contributions presented in this study are included in the article/Supplementary Material. Further inquiries can be directed to the corresponding authors.

## Supplementary Materials

The following supporting information can be downloaded at: https://www.mdpi.com/article/10.3390/vetsci13101029/s1, Table S1: Annual sampling and isolation of *K. pneumoniae* from calves with BRD in Henan Province, China, 2021–2026. Table S2: Primers of resistance genes. Table S3: Primers of virulence-associated genes. Table S4: Antimicrobial resistance genes distributions among 87 strains.
