# Supplementary material for: Isolation Rate, Antimicrobial Resistance, Virulence-Associated Genes, and Multilocus Sequence Types of Klebsiella pneumoniae Isolated from Cattle with Bovine Respiratory Disease in Henan Province, China
Source: Vet Sci. 2026 Sep 29;13(10):1029. doi: 10.3390/vetsci13101029 (PMC13709525; doi:10.3390/vetsci13101029)
Supplement: Supplementary file 1 [file vetsci-13-01029-s001.zip › vetsci-4572516-supplementary.pdf]

Table S1 Annual sampling and isolation of K. pneumoniae from calves with BRD in Henan Province, China, 2021–2026.

Panel A. Annual sample sizes, positive counts, and isolation rates.

Year	Sample period	No. of farms	No. of cities	No. of Samples	No. of positive isolates	Isolation rate % (95% CI)
2021	Apr-Dec	3	2	46	8	17.4 (9.1–30.7)
2022	Mar-Oct	4	4	50	8	16.0 (8.3–28.5)
2023	Feb-Dec	8	6	114	21	18.4 (12.4–26.5)
2024	Jan-Dec	8	5	116	23	19.8 (13.6–28.0)
2025	Jan-Dec	10	7	130	18	13.8 (8.9–20.8)
2026	Jan-Jun	5	4	61	9	14.8 (8.0–25.7)
Total		38	13	517	87	16.8 (13.9–20.3)

Panel B. Sampling framework and animal characteristics.

Characteristic	Category	n (%) or description
Age range	1–6 months	Median 3 months; IQR 2–4 months
Sex	Male	270 (52.2%)
	Female	247 (47.8%)
Production type	Intensive	10 farms
	Semi-intensive	17 farms
	Backyard	11farms
Prior antimicrobial exposure	Yes, within 14 days before sampling	14 farms (36.8%)
	No	22 farms (57.9%)
	Unknown	2 farms (5.3%)
Farm selection criteria	≥20 calves, history of BRD, owner consent	—
Animal selection criteria	Wisconsin score ≥5, no prior sampling, no other obvious systemic disease	—
Sampling period within each year	Varied by year (see Table S1A)	—
Repeated sampling	None	Each calf sampled once

Table S2 Primers of resistance genes.

Gene name	Sequence (5′-3′)	Product size (bp)	Annealing temperature (℃)	Reference
blaVIM	F: GATGGTGTTTGGTCGCATA<br>R: CGAATGCGCAGCACCAG	390	55	Candan et al., 2015
blaSHV	F: ATGCGTTATATTCGCCTGTG<br>R: TGCTTTGTTATTCGGGCCAA	747	55	Zhang et al., 2016
blaTEM	F: TCGCCGCATACACTATTCTCAGAATGA<br>R: ACGCTCACCGGCTCCAGATTTAT	445	55	Zhang et al., 2016
blaCTX-M-2	F: AAATGTGCTGCTCCTTTCGTGAGC<br>R: AGGGTTCGTTGCAAGACAAGACTG	1122	55	Andrade et al., 2018
sul1	F: GTGACGGTGTTCGGCATTCT<br>R: TCCGAGAAGGTGATTGCGCT	779	55	Fazel et al., 2019
sul 2	F: CGGCATCGTCAACATAACCT<br>R: TGTGCGGATGAAGTCAGCTC	721	55	Fazel et al., 2019
sul 3	F: CATTCTAGAAAACAGTCGTAGTTC<br>R: CATCTGCAGCTAACCTAGGGCTTTGGA	973	55	Fazel et al., 2019
tetA	F: GCCTTTCCTTTGGGTTCTCT<br>R: TGTCCGACAAGTTGCATGAT	402	55	Chiu et al., 2017
tetB	F: CCTCAGCTTCTCAACGCGTG<br>R: GCACCTTGCTGATGACTCTT	634	55	Tabaran et al., 2017
tetC	F: ACTTGGAGCCACTATCGAC<br>R: CTACAATCCATGCCAACCC	880	50	Maynard et al., 2004
cat Ⅰ	F: AGTTGCTCAATGTACCTATAACC<br>R: TTGTAATTCATTAAGCATTCTGCC	547	50	Maynard et al., 2003
cmlA	F: TTGCAACAGTACGTGACAT<br>R: ACACAACGTGTACAACCAG	293	55	Travis et al., 2006
floR	F: CGCCGTCATTCCTCACCTTC<br>R: GATCACGGGCCACGCTGTGTC	215	50	Maynard et al., 2003
aadA1	F: TATCCAGCTAAGCGCGAAC<br>R: ATTTGCCGACTACCTTGGT	447	55	Tabaran et al., 2017
aacC1	F: ATGGGCATCATTCGCACATGTAGG<br>R: TTAGGTGGCGGTACTTGGGTC	861	55	Al-Marzooq et al., 2015
aacC2	F: ATGCATACGCGGAAGGCAATAAC<br>R: CTAACCGGAAGGCTCGCAAG	861	55	Al-Marzooq et al., 2015
oqxA	F: CCACTCTTCACGGGAGACGA<br>R: TTCTCCCCCGGCGGGAAGTAC	392	55	Rodríguez-Martínez et al., 2013
aac(6′)-Ib-cr	F: TTGCGATGCTCTATGAGTGGCTA<br>R: CTCGAATGCCTGGCGTGTTT	482	55	Park et al., 2006
qnrA	F: TCAGCAAGCGGATTTCTCA<br>R: GGCAGCACTATTACTCCCA	608	55	Yang et al., 2019
qnrB	F: GATCGTGAAAGCCAGAAAGG	469	53	

	R: ACGATGCCTGGTAGTTGTCC			Shin et al., 2008
qnrS	F: ACGACATTCGTCAACTGCAA<br>R: TAAATTGGCACCCTGTAGGC	417	53	Shin et al., 2008
gyrA	F: ACGTACTAGGCAATGACTGG<br>R: AGAAGTCGCCGTCGATAGAAC	189	55	Ko et al., 2009

Table S3 Primers of virulence genes.

Gene name	Sequence (5′-3′)	Product size (bp)	Annealing temperature (℃)	Reference
fimH	F: TGCTGCTGGGCTGGTCGATG<br>R: GGGAGGGTGACGGTGACATC	688	55	Xu et al., 2019
mrkD	F: AAGCTATCGCTGTACTTCCGGCA<br>R: GGCGTTGGCGCTCAGATAGG	340	55	Lan et al., 2019
uge	F: TCTTCACGCCTTCCTTCACT<br>R: GATCATCCGGTCTCCCTGTA	534	53	Osman et al., 2014
wabG	F: ACCATCGGCCATTTGATAGA<br>R: CGGACTGGCAGATCCATATC	683	53	Xu et al., 2019
entB	F: ATTTCCTCAACTTCTGGGGC<br>R: AGCATCGGTGGCGGTGGTCA	371	60	Candan et al., 2015
iutA	F: AGGCTTCGTCACAGTTG<br>R: CCATCGTCACCAGAGGA	570	60	Sivaraman et al., 2020
iroN	F: AAGTCAAAGCAGGGGTTGCCCG<br>R: GATCGCCGACATTAAGACGCAG	667	55	Rodriguez-Siek et al., 2005
kfu	F: GAAGTGACGCTGTTTCTGGC<br>R: TTTCGTGTGGCCAGTGACTC	797	55	Osman et al., 2014
ureA	F: GCTGACTTAAGAGAACGTTATG<br>R: GATCATGGCGCTACCT(C/T)A	337	55	Brisse et al., 2009
allS	F: CCGAAACATTACGCACCTTT<br>R: ATCACGAAGAGCCAGGTCAC	508	49	Xu et al., 2019
terB	F: TATCGCTGTTGCCAGTGAC<br>R: AATACCGACACGAAGAGCC	235	53	Harada et al., 2018
hly	F: AACAAGGATAAGCACTGTTCTGGCT<br>R: ACCATATAAGCGGTCATTCCCGTCA	1177	58	Candan et al., 2015
rmpA	F: GTAATAGAGATATAAATATCATATTGA<br>R: CATCTTTCATCAACCATTTC	588	50	Harada et al., 2018
rmpA2	F: GTGCAATAAGGATGTTACATTA<br>R: GGATGCCCTCCTCCTG	430	50	Harada et al., 2018

Table S4 Antimicrobial resistance genes distributions among 87 strains

Strains/Antibiotic resistance genes	*blaTEM*	*blaSHV*	*sul1*	*sul2*	*tetA*	*tetB*	*tetC*	*floR*	*aadA1*	*qnrB*	*qnrS*
1	+[a]	-[b]	-	+	-	-	-	-	-	-	-
2	+	-	-	+	-	-	-	-	-	-	-

3	-	-	-	-	-	-	+	-	-	-	-
4	-	-	+	-	-	-	-	-	-	+	-
5	+	+	-	-	-	-	+	-	-	+	-
6	+	+	+	-	-	-	-	-	-	-	-
7	+	+	+	-	-	-	-	-	-	-	-
8	+	+	-	-	-	-	-	-	-	-	-
9	+	-	-	-	+	-	-	-	-	-	-
10	-	+	+	-	-	-	-	-	-	-	-
11	-	-	+	-	-	-	-	-	+	-	-
12	+	-	-	-	-	-	-	-	-	-	-
13	+	+	-	+	-	-	-	-	-	-	-
14	+	+	-	+	-	-	-	-	-	-	-
15	+	+	-	-	-	-	+	-	-	-	-
16	+	-	+	-	-	-	-	-	-	-	-
17	+	-	+	-	-	-	-	-	-	-	-
18	+	-	-	-	-	-	-	-	-	-	-
19	-	-	-	-	-	-	-	+	-	+	-
20	-	+	+	-	-	-	-	-	+	-	-
21	-	+	+	-	-	-	-	-	+	-	-
22	+	-	-	-	-	-	+	-	-	-	-
23	+	-	-	-	-	-	-	-	-	+	-
24	+	-	-	-	-	-	-	-	-	-	-
25	+	+	-	+	-	-	-	-	-	-	-
26	-	-	+	-	-	-	-	-	-	-	-
27	-	-	+	-	-	-	-	-	-	-	-
28	+	-	-	-	-	-	-	-	-	-	-
29	+	+	-	-	-	-	-	-	-	-	-
30	+	+	-	-	-	-	-	-	-	-	-
31	+	+	-	-	-	-	-	-	-	-	-
32	+	+	-	-	-	-	-	-	-	-	-
33	+	+	-	-	-	-	-	-	-	-	+
34	+	+	+	-	-	-	-	-	-	-	-
35	+	+	+	-	-	-	-	-	-	-	-
36	+	-	+	-	-	-	-	-	-	-	-
37	+	-	+	-	-	-	-	-	-	-	-
38	-	-	+	-	-	-	-	-	-	-	-
39	-	-	+	-	-	-	-	-	-	-	-
40	-	-	+	-	-	-	+	-	-	+	-
41	-	-	-	-	+	-	-	-	-	+	-
42	-	-	-	-	+	-	-	-	-	+	-
43	+	+	+	-	-	-	-	-	-	-	-
44	+	+	-	+	-	-	-	-	-	-	-
45	+	+	-	+	-	-	-	-	-	-	-
46	+	+	-	+	-	-	-	-	-	-	-

47	+	-	-	-	-	-	-	-	-	-	-
48	+	-	-	-	-	-	-	-	-	-	-
49	+	-	-	-	-	-	-	-	-	+	-
50	+	-	-	-	-	-	-	-	-	+	-
51	+	+	-	+	-	-	+	-	-	-	-
52	+	+	-	-	-	-	-	-	-	-	-
53	+	-	+	-	-	-	-	-	-	-	-
54	+	-	-	-	-	-	-	-	+	-	-
55	+	-	-	-	-	-	-	-	+	-	-
56	+	-	-	+	-	-	-	-	+	-	-
57	+	-	-	+	-	-	-	-	-	-	-
58	-	-	+	-	-	-	-	-	-	-	-
59	-	-	+	-	-	-	+	-	-	-	-
60	-	-	+	-	-	-	+	-	+	-	-
61	+	-	+	-	-	-	-	-	-	-	-
62	+	-	+	-	-	-	-	-	-	-	-
63	+	-	+	-	-	-	-	-	-	-	-
64	-	-	+	-	-	-	+	-	-	-	-
65	-	-	+	-	-	-	-	-	-	-	-
66	-	-	+	+	-	-	-	-	-	+	-
67	+	-	-	+	-	-	-	-	-	+	-
68	+	-	-	-	-	-	-	-	-	-	-
69	+	+	-	-	-	-	-	-	+	-	-
70	+	+	-	-	-	-	-	-	-	-	-
71	+	+	-	-	-	-	-	-	-	-	-
72	+	-	-	-	-	-	-	+	-	-	-
73	+	+	-	-	-	-	-	-	-	-	-
74	+	-	-	-	-	+	-	-	-	+	-
75	+	-	-	-	-	+	-	-	-	+	-
76	+	-	-	-	-	-	-	-	-	-	-
77	-	-	-	-	-	+	-	-	+	-	-
78	-	-	-	-	-	+	-	-	-	-	-
79	+	+	-	+	-	-	-	-	-	-	-
80	+	+	-	+	-	-	-	-	-	-	-
81	+	+	-	+	-	-	-	-	-	-	-
82	+	-	-	-	-	-	-	-	-	-	-
83	+	-	+	-	-	-	-	-	-	-	-
84	-	-	+	-	-	-	+	-	-	-	-
85	-	-	+	-	-	-	+	-	-	-	-
86	+	+	+	-	-	-	-	-	-	-	-
87	+	+	+	-	-	-	-	-	-	-	-

[a]:+: positive for detected gene.

[b]: -: negative for detected gene.
